# Supplementary material for: Staphylococcus aureus induces an itaconate-dominated immunometabolic response that drives biofilm formation
Source: Nat Commun. 2021 Mar 3;12:1399. doi: 10.1038/s41467-021-21718-y (PMC7930111; doi:10.1038/s41467-021-21718-y)
Supplement: Supplementary file 1 — Supplementary Information [file 41467_2021_21718_MOESM1_ESM.pdf]

SUPPLEMENTARY INFORMATION

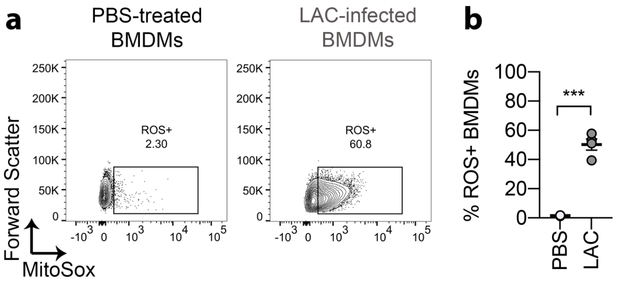

Supplementary Figure 1: *S. aureus* induces ROS production in primary cells. a, b. Mitochondrial ROS (MitoSox) in bone marrow-derived macrophages (BMDMs) treated with PBS or infected with LAC. Data are shown as mean from n=3 biological replicates from one independent experiment. Significance determined by two-tailed t-Student: ***: $P < 0.001$.

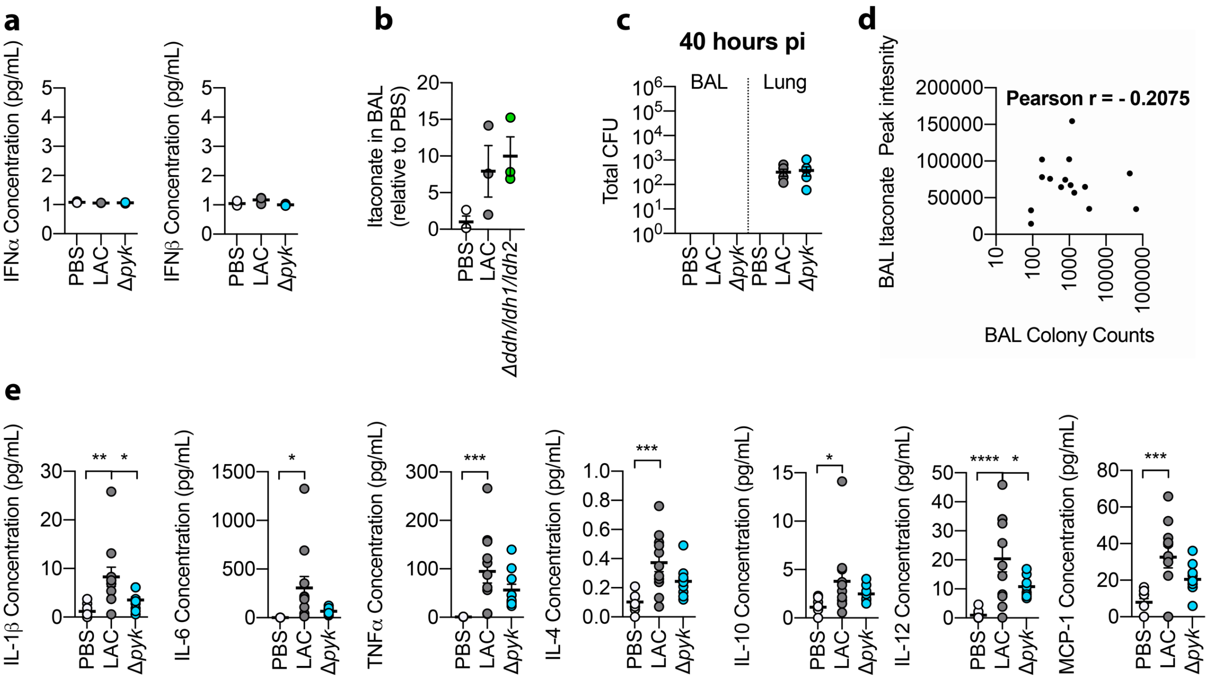

Supplementary Figure 2: *S. aureus* glycolytic activity is not essential for survival in the airway but does impact the host immune response. a. Type I interferons in the BAL fluid of mice treated with PBS or infected with LAC or Δ*pyk* 16 hours post infection. **b.** Itaconate in the BAL fluid of mice treated with PBS or infected with WT LAC or a lactate-deficient mutant (Δ*ddh/ldh1/ldh2*). **c.** Total colony forming units (CFU) in the BAL fluid and lung tissue of mice treated with PBS or infected with LAC or Δ*pyk* 40 hours post infection (pi). **d.** Correlation of itaconate levels and colony counts in the BAL of LAC and Δ*pyk*-infected mice. **e.** Cytokines in the BAL fluid of mice treated with PBS or infected with LAC or Δ*pyk* 16 hours post infection. Data are shown as mean ± SEM from n=3 (a, b), 6 (c) or 11 (e) mice. Significance determined by One-Way ANOVA with Tukey's Multiple Comparisons (e); *: $P < 0.05$, **: $P < 0.01$, ***: $P < 0.001$, ****: $P < 0.0001$.

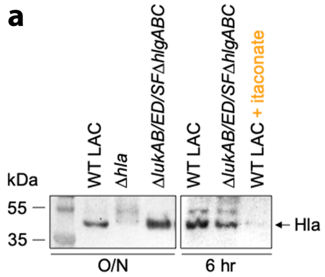

Supplementary Figure 3: Itaconate suppresses alpha toxin production. a. Hla toxin production by WT LAC, toxin-deficient mutants (Δ*hla* and Δ*lukAB/ED/SF*Δ*hlgABC*), and WT LAC exposed to itaconate (30 mM).

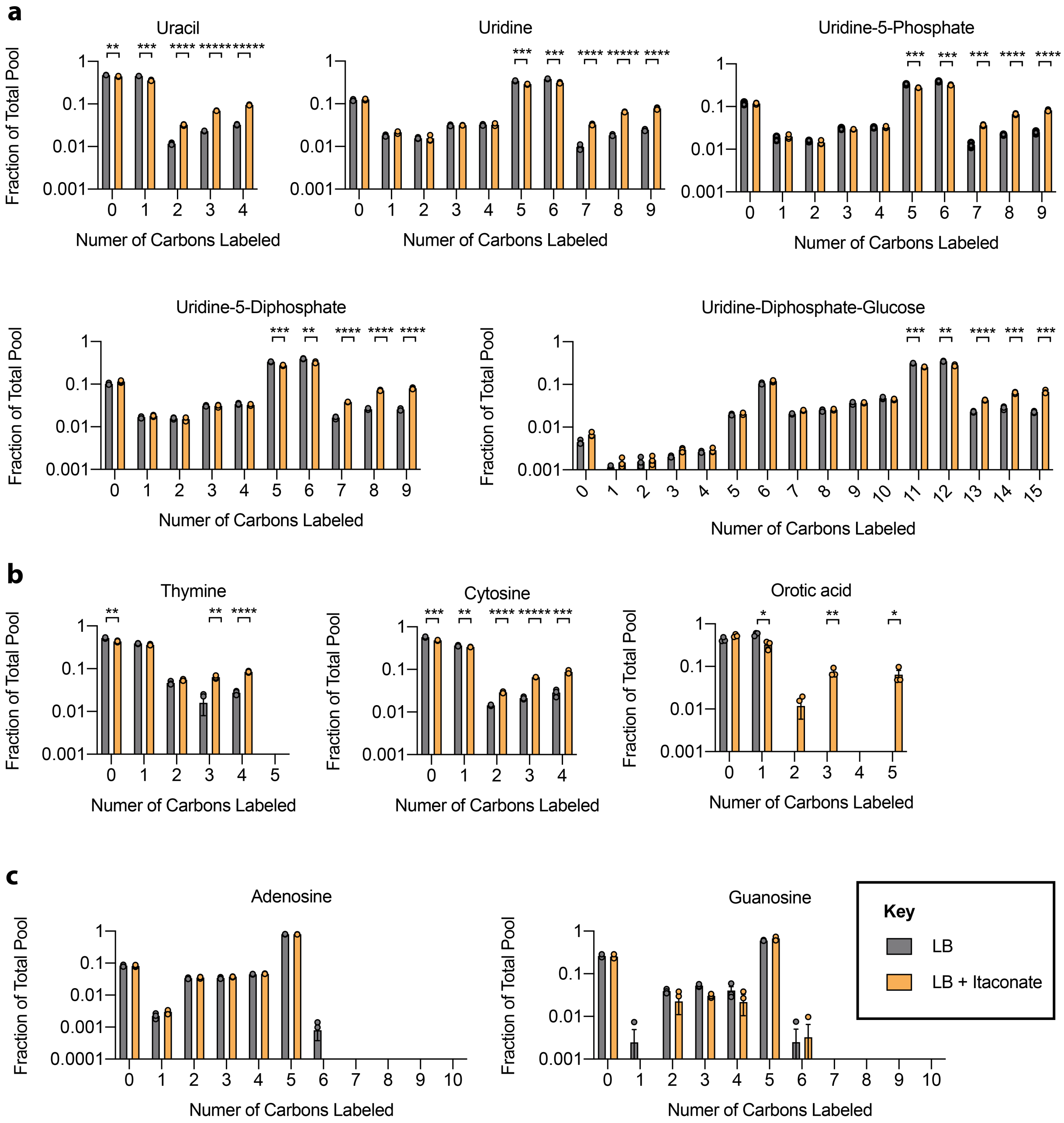

Supplementary Figure 4: Itaconate increases carbon flux through pyrimidine synthesis pathways. ^{13}C-glucose labeling of LAC metabolites involved in **a.** UTP synthesis, **b.** pyrimidine synthesis, and **c.** purine synthesis in the presence or absence of itaconate (30 mM). For each molecule, the different isotopologues are shown. Data are shown as mean ± SEM from n=3 biological replicates from one independent experiment. Significance determined by two-tailed t-Student with FDR correction; *: $P < 0.05$, **: $P < 0.01$, ***: $P < 0.001$, ****: $P < 0.0001$, *****: $P < 0.00001$.

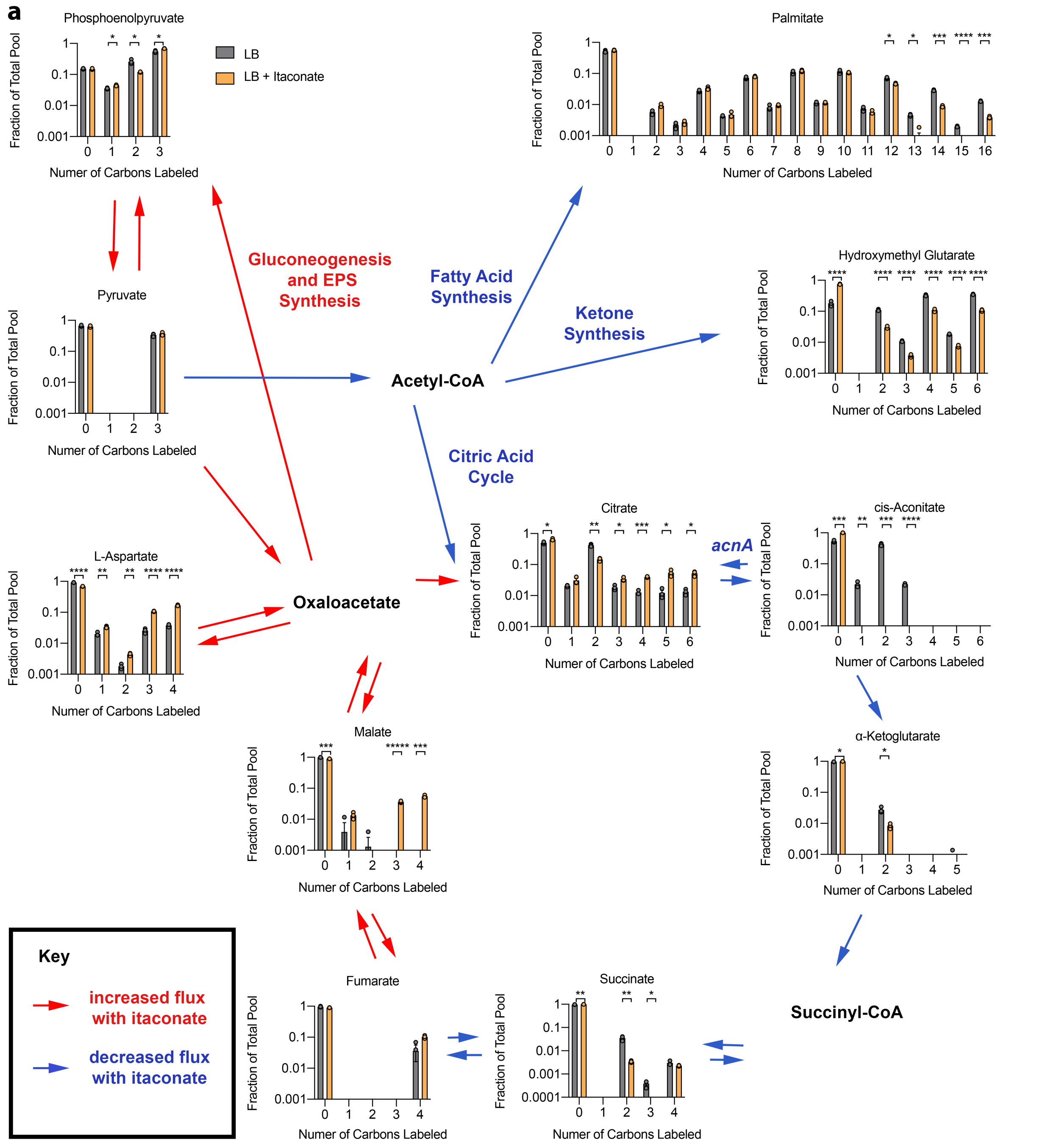

Supplementary Figure 5: Itaconate increases carbon flux through gluconeogenic precursors and decreases flux through competing pathways. a. ^{13}C-glucose labeling of LAC metabolites involved in central carbon metabolism in the presence or absence of itaconate (30 mM). For each molecule, the different isotopologues are shown. Data are shown as mean ± SEM from n=3 biological replicates from one independent experiment. Significance determined by two-tailed t-Student with FDR correction; *: $P < 0.05$, **: $P < 0.01$, ***: $P < 0.001$, ****: $P < 0.0001$, *****: $P < 0.00001$.

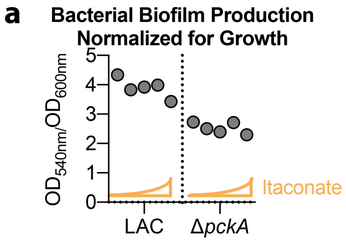

Supplementary Figure 6: Biofilm production depends on flux through *pckA*. a. Biofilm production (normalized for growth) of WT LAC and a Δ*pckA* mutant in the presence of the gluconeogenic precursor pyruvate (0.5%) in increasing itaconate concentrations (0 to 62 mM). Data are shown as mean from n=2 biological replicates from two independent experiments.

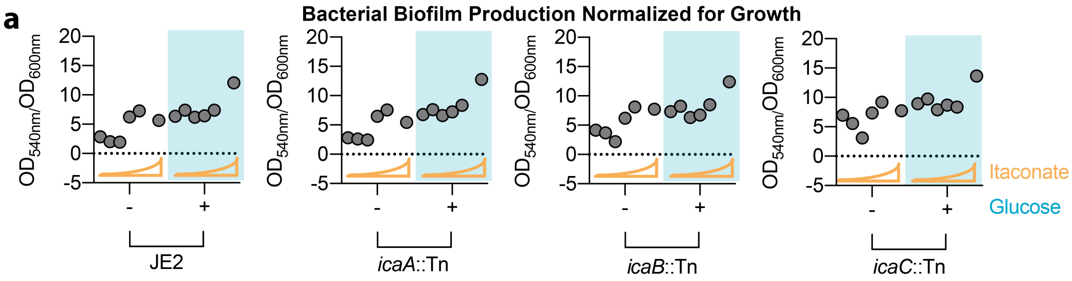

Supplementary Figure 7: Itaconate-driven biofilm production is not *ica*-dependent. a. Biofilm production (normalized for growth) of JE2 and *ica* transposon mutants in increasing itaconate concentrations (0 to 62 mM), with or without glucose (0.5%). Data are shown as mean from n=2 biological replicates from two independent experiments.

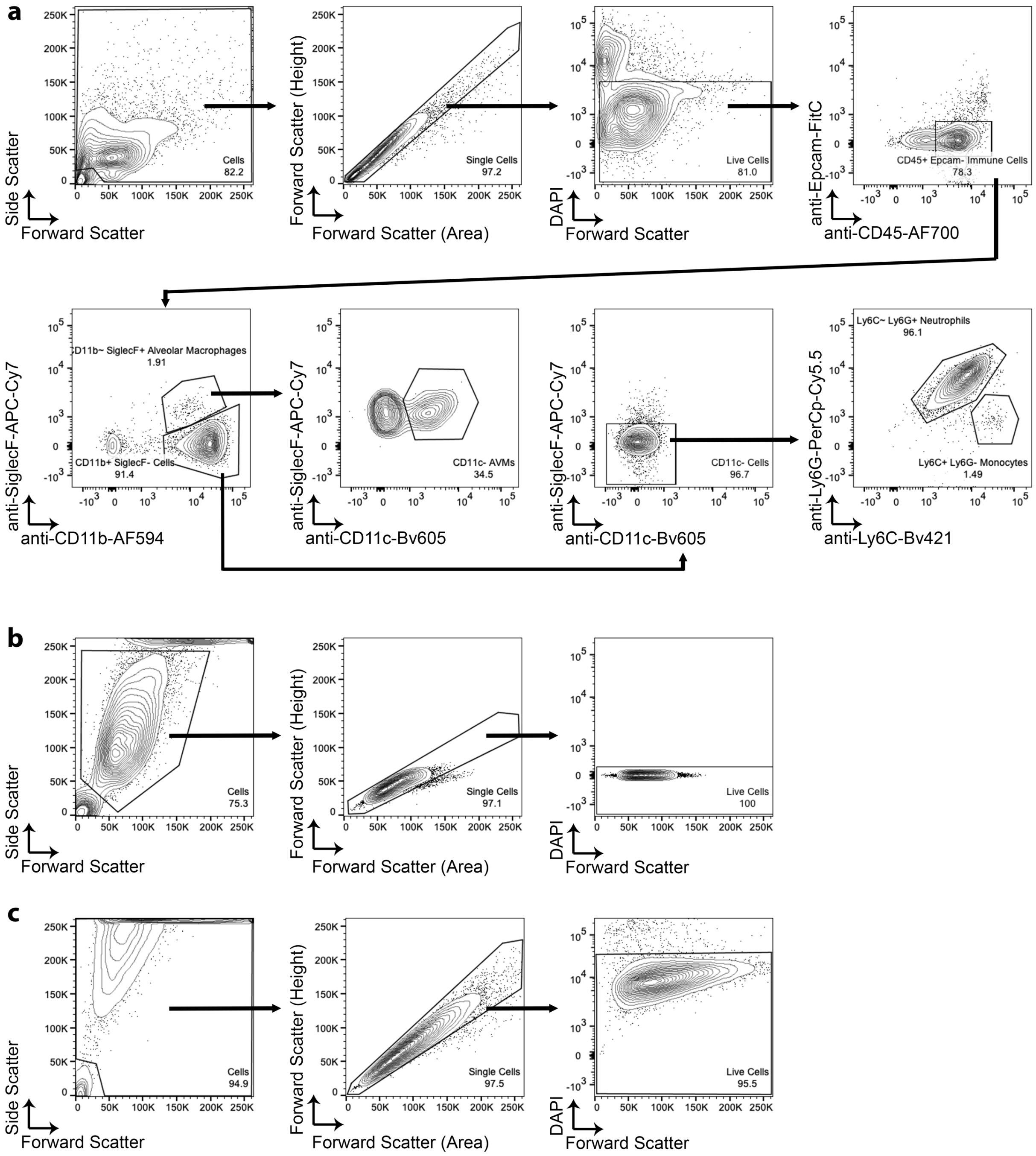

Supplementary Figure 8: Gating strategies for FACS analyses in this study. a. Identification of mouse BAL and lung immune cells (as seen in Figures 1c-d, 2e-f and 2i). **b.** Mitochondrial depolarization and ROS detection in THP1-cells (as seen in Figures 1g-h and 2b-d). **c.** IRG1 detection in THP-1 cells (as seen in Figure 1i).

Supplementary Table 1: Bacterial strains used in this study.

S. aureus Strain	Source	Reference
USA300 LAC	Anthony Richardson, University of Pittsburgh, USA	[22]
Δ*pyk* LAC	Anthony Richardson, University of Pittsburgh, USA	[22]
Δ*ddh/ldh1/2* LAC	Tammy Kielian, University of Nebraska, USA	[37]
Δ*hla* LAC	Juliane Bubeck-Wardenburg, Washington University, USA	[55]
Δ*lukAB/ED/SF*/ Δ*hlgABC* LAC	Victor Torres, New York University, USA	Lab collection
JE2	Nebraska Transposon Mutant Library	Public collection
Tn::*icaA*	Nebraska Transposon Mutant Library	Public collection
Tn::*icaB*	Nebraska Transposon Mutant Library	Public collection
Tn::*icaC*	Nebraska Transposon Mutant Library	Public collection
A1 (ST105)	Jonathan Koff, Yale University, USA	[19] (Clinical isolate)
A5 (ST105*)	Jonathan Koff, Yale University, USA	[19] (Clinical isolate)
A6 (ST105*)	Jonathan Koff, Yale University, USA	[19] (Clinical isolate)
A 2001 (ST30)	Barbara Kahl, University Hospital Münster, Germany	This study (clinical isolate)
D 2003 (ST30)	Barbara Kahl, University Hospital Münster, Germany	This study (clinical isolate)
G 2007 (ST30)	Barbara Kahl, University Hospital Münster, Germany	This study (clinical isolate)
L 2011 (ST30)	Barbara Kahl, University Hospital Münster, Germany	This study (clinical isolate)
N 2013 (ST30**)	Barbara Kahl, University Hospital Münster, Germany	This study (clinical isolate)
O 2014 (ST30)	Barbara Kahl, University Hospital Münster, Germany	This study (clinical isolate)
T 2015 (ST30)	Barbara Kahl, University Hospital Münster, Germany	This study (clinical isolate)

* SNP in *pta*

** different allele in *arcC*

Supplementary Table 2: Primers used in this study.

Locus	Forward Primer sequence 5'-3'	Reverse Primer sequence 5'-3'
agrA	CAAAGTTGCAGCGATGGATTT	AGCGTGTATGTGCAGTTTCT
sarR	TTTAGTCAACGCAACATTTCAA	GGTTTGAACTCTGAGCACTTAGC
hla	GGCTCTATGAAAGCAGCAGATA	CTGTAGCGAAGTCTGGTGAAA
splA	CATTCAATTGCCAAAGCAGA	TTTCCTCCGCCTTTACCTTT
ear	GGCGGTGAAAACAGTATTGAA	CACCGTCTTTCACAATCTGC
perR	AATTGCATCATTGCGACAAG	TGGAAAATCAGGTGAAAGTGC
rplB	TACTTGCCGTGCTACAATCG	CAGAACCACGAACTGTTGGA
rplW	GCAATGGCTGAAGACAAATACA	GTAACGGCCCATACGTTTTTC
dnaK	ACAAACTTGGCGGTGATGAT	TCAGCAGCATCTTTCAAACG
clpB	AGACAAGCGTTGGAAGATGC	ATCGCCAATTTCTTCGTCTG
topA	GGGAAACAAGGTGACCAAGA	GCAACAAATCGTTCCCAAAT
relA	ATTAGACGGACCGACGATTG	TTGCGATGATTTTCAGCTTG
pyc	GGCCAAGAAGCACTAACAGC	TTTCACCATTACGCATTCCA
pckA	AATCGGCGGTACTGAATACG	AGTTTACGGTGTGGGTCAGC
pyk	TGCAGCAAGTTTCGTACGTC	TTCAACACCCATGTCACCAC
fba	GCCCATTCGAAGAAAACGTA	ACCTAATGCTGGCGCTAATG
ldh1	GGTGTTGCAATGGGATTAGC	TGTGCGAACTTGCTTTGTTC
ldh2	CGGCAAACCCTGTAGACATT	TATCACCATGCTCGCCAATA
acnA	GCGCAACAGCAACTGATTTA	TAGCGCGATATGTTCGTCTG
icaA	ACACTTGCTGGCGCAGTCAA	TCTGGAACCAACATCCAACA
icaB	TCCTTATGGCTTGATGAATGACG	CTAATCTTTTTCATGGAATCCGTCC
icaC	ATGGGTTATAACTACGAACGTG	CGTGCAAATACCCAAGATAAC
icaD	ATGGTCAAGCCCAGACAGAG	AGTATTTTCAATGTTTAAAGCAA
