# Supplementary material for: Staphylococcus aureus induces an itaconate-dominated immunometabolic response that drives biofilm formation
Source: Nat Commun. 2021 Mar 3;12:1399. doi: 10.1038/s41467-021-21718-y (PMC7930111; doi:10.1038/s41467-021-21718-y)
Supplement: Supplementary file 3 — Reporting Summary [file 41467_2021_21718_MOESM3_ESM.pdf]

nature research

Corresponding author(s): Sebastian A. Riquelme

Last updated by author(s): Jan 26, 2021

Reporting Summary

Nature Research wishes to improve the reproducibility of the work that we publish. This form provides structure for consistency and transparency in reporting. For further information on Nature Research policies, see our Editorial Policies and the Editorial Policy Checklist.

Statistics

For all statistical analyses, confirm that the following items are present in the figure legend, table legend, main text, or Methods section.

n/a	Confirmed	
☐	☒	The exact sample size (*n*) for each experimental group/condition, given as a discrete number and unit of measurement
☐	☒	A statement on whether measurements were taken from distinct samples or whether the same sample was measured repeatedly
☐	☒	The statistical test(s) used AND whether they are one- or two-sided<br>*Only common tests should be described solely by name; describe more complex techniques in the Methods section.*
☒	☐	A description of all covariates tested
☐	☒	A description of any assumptions or corrections, such as tests of normality and adjustment for multiple comparisons
☐	☒	A full description of the statistical parameters including central tendency (e.g. means) or other basic estimates (e.g. regression coefficient) AND variation (e.g. standard deviation) or associated estimates of uncertainty (e.g. confidence intervals)
☐	☒	For null hypothesis testing, the test statistic (e.g. *F*, *t*, *r*) with confidence intervals, effect sizes, degrees of freedom and *P* value noted<br>*Give P values as exact values whenever suitable.*
☒	☐	For Bayesian analysis, information on the choice of priors and Markov chain Monte Carlo settings
☒	☐	For hierarchical and complex designs, identification of the appropriate level for tests and full reporting of outcomes
☒	☐	Estimates of effect sizes (e.g. Cohen's *d*, Pearson's *r*), indicating how they were calculated

Our web collection on statistics for biologists contains articles on many of the points above.

Software and code

Policy information about availability of computer code

Data collection	BD FACSDiva v9 was used for acquisition of the flow cytometry data. Seahorse Wave Desktop v2.6.0 was used for acquisition of the extracellular flux data. StepOne v2.3 was used for acquisition of the qRT-PCR data. Tecan iControl v1.10.4 was used for acquisition of the growth curves, biofilm and carbon source assimilation data. SoftMax Pro v7.0.3 was used for acquisition of the aldolase activity assay data. E-MAVEN v0.10.0 and MAVEN 2011.6.17 were used for acquisition of metabolomics data.
Data analysis	E-MAVEN v0.10.0 and MAVEN 2011.6.17 were used to quantify metabolite signals. FlowJo v10 was used for cell gating and signal quantification. Porechop v0.2.4, Mothur v1.22.2, SPAdes v3.10.1, and Prokka v1.12 were used to trim, filter, assemble, and annotate nanopore reads, respectively. STAR-Aligner v2.7.3a, GATK Picard tools v2.22.3, Subreads:FeatureCounts v1.6.3, and DESeq2 in R v3.5.3 were used to align, annotate, quantify, and analyze differential expression of RNA-seq reads, respectively.

For manuscripts utilizing custom algorithms or software that are central to the research but not yet described in published literature, software must be made available to editors and reviewers. We strongly encourage code deposition in a community repository (e.g. GitHub). See the Nature Research guidelines for submitting code & software for further information.

Data

Policy information about availability of data

All manuscripts must include a data availability statement. This statement should provide the following information, where applicable:

- Accession codes, unique identifiers, or web links for publicly available datasets
- A list of figures that have associated raw data
- A description of any restrictions on data availability

All data discussed in this study are presented in the published article and its supplementary files, which also include a reporting summary. Source data are provided as a supplementary file, but are also are available upon request to the contributing author. Genomic data and transcriptomic data are available in the Sequence

Read Archive, under BioProject accession PRJNA686110 (www.ncbi.nlm.nih.gov/bioproject/PRJNA686110) (Figures 3a and 5a). Metabolomic data are available in the MetaboLights database, under accession MTBLS2405 (www.ebi.ac.uk/metabolights/MTBLS2405) (Figures 4a and Supplementary Figures 4-5).

Field-specific reporting

Please select the one below that is the best fit for your research. If you are not sure, read the appropriate sections before making your selection.

☒ Life sciences ☐ Behavioural & social sciences ☐ Ecological, evolutionary & environmental sciences

For a reference copy of the document with all sections, see nature.com/documents/nr-reporting-summary-flat.pdf

Life sciences study design

All studies must disclose on these points even when the disclosure is negative.

Sample size	Sample sizes were determined by power analyses based on detecting effect sizes observed in previous experiments with a power of 0.8 at a significance threshold of 0.05 for the given statistical test.
Data exclusions	A few data points were excluded based on pre-established criteria using the ROUT detection method with a Q = 1%. These include 2 data points in the correlation analysis in Supplemental Figure 2D.
Replication	To ensure the reproducibility of the data, both technical replicates and biological replicates were used. Biological replicates were derived from biologically independent mice, cell cultures, and/or experiments (performed on different days, with different samples and controls). The details for how often independent experiments were performed for are provided in the figure legends. For each experiment, all attempts at replication were successful.
Randomization	Samples and animals were randomly allocated into experimental groups.
Blinding	There was no blinding during data acquisition or analysis in this study.

Reporting for specific materials, systems and methods

We require information from authors about some types of materials, experimental systems and methods used in many studies. Here, indicate whether each material, system or method listed is relevant to your study. If you are not sure if a list item applies to your research, read the appropriate section before selecting a response.

Materials & experimental systems

n/a	Involved in the study	
☐	☒	Antibodies
☐	☒	Eukaryotic cell lines
☒	☐	Palaeontology and archaeology
☐	☒	Animals and other organisms
☐	☒	Human research participants
☒	☐	Clinical data
☒	☐	Dual use research of concern

Methods

n/a	Involved in the study	
☒	☐	ChIP-seq
☐	☒	Flow cytometry
☒	☐	MRI-based neuroimaging

Antibodies

Antibodies used	Antibodies used in this study include: anti-CD45-AF700 (BioLegend cat# 103127, lot# B274308), anti-CD11b-AF594 (BioLegend cat# 101254, lot# B256615), anti-CD11c-Bv605 (BioLegend cat# 117334, lot# B292874), anti-SiglecF-APC-Cy7 (BD Biosciences cat# 565527, lot# 7244817), anti-Epcam-FITC (BioLegend #118207), anti-F4/80-Pe-Cy7 (BioLegend cat# 123114, lot# B237342), anti-Ly6C-Bv421 (BioLegend cat# 128032, lot# B250487), anti-Ly6G-PerCp-Cy5.5 (BioLegend cat# 127616, lot# B248844), anti-Irg1 (Abcam cat# ab222411, lot# GR3237083-14), anti-rabbit-AF647 (Invitrogen cat# A31573, lot# 1964354), anti-hla (Sigma cat# S7531), and anti-rabbit-horseradish peroxidase (Abcam cat# SC2357).
Validation	Target specificity and functional validation for each antibody was performed by the manufacturer, and validation statements for each antibody can be found on the manufacturer's website.

Eukaryotic cell lines

Policy information about cell lines

Cell line source(s)	The THP-1 cell line was sourced from the ATCC (TIB-202) and has been expanded to working stocks within our lab.
Authentication	The THP-1 cell line was authenticated by STR profiling by the supplier.

Mycoplasma contamination	The THP-1 cell line was negative for mycoplasma upon receipt from the supplier and when expanded to working stocks.
Commonly misidentified lines (See ICLAC register)	No commonly misidentified cell lines were used in this study.

Animals and other organisms

Policy information about studies involving animals; ARRIVE guidelines recommended for reporting animal research

Laboratory animals	In this study, 7-9 week-old, mixed-sex mice (WT C57Bl6N, stock number 000664, from Jackson Laboratories) were used. These mice were housed in humidity-controlled conditions at 18-23 degrees Celsius, with 12 hour light/dark cycles.
Wild animals	No wild animals were used in this study.
Field-collected samples	No field-collected samples were used in this study.
Ethics oversight	All animal studies were subject to oversight by the Columbia Institutional Animal Care and Use Committee (IACUC) and were approved under protocols AAAR9406 and AABE8600.

Note that full information on the approval of the study protocol must also be provided in the manuscript.

Human research participants

Policy information about studies involving human research participants

Population characteristics	Sputum was collected from 7 CF and 5 healthy control subjects (22-44 years of age). An informed consent was signed by all subjects providing samples. All CF samples were positive for S. aureus respiratory infection. Male and female adult samples were collected in an approximate 50%–50% ratio. Under the clinical settings of this study, age and gender are not expected to influence the variables tested.
Recruitment	Sputum samples from healthy adult individuals and subjects with CF were provided by Dr. Emily DiMango from Columbia University Medical Center, and Dr. Clemente Britto-Leon from Yale University (IRB AAAR1395 and 0102012268). Patients were invited to participate during routine medical visits. The project and protocols were carefully explained by their primary physician. If patients chose to participate, they signed consent forms approved by the same IRB protocols mentioned above. For protection of personal information, these forms are maintained under a double locked cabinet by their primary physician.<br><br>Given that participation is voluntary, there may be self-selection bias in the recruitment of these subjects, i.e. individuals with increased infection and inflammation could be more likely to participate in routine medical visits and be enrolled in this study. However, this self-selection bias likely did not influence the results of our analysis, given that the outcome being measured (itaconate concentration) was linked to infection status, which was objectively determined by clinical cultures.
Ethics oversight	Human Samples collections protocols and usage of experimental information are approved under Columbia IRB Protocols AAAR1395 and Yale IRB Protocol 0102012268. Private patient information is protected under these permits.

Note that full information on the approval of the study protocol must also be provided in the manuscript.

Flow Cytometry

Plots

Confirm that:

- [x] The axis labels state the marker and fluorochrome used (e.g. CD4-FITC).
- [x] The axis scales are clearly visible. Include numbers along axes only for bottom left plot of group (a 'group' is an analysis of identical markers).
- [x] All plots are contour plots with outliers or pseudocolor plots.
- [x] A numerical value for number of cells or percentage (with statistics) is provided.

Methodology

Sample preparation	For the cells harvested from infected mice: bronchoalveolar lavage fluid (BAL) was collected by intratracheal lavage and lung tissue was collected and homogenized through 40 um cell strainers. Red blood cells were lysed hypotonically in ACK lysis buffer, and the remaining cells were extracelullarly stained in buffered saline, fixed, permeabilized, and intracellularly stained using the eBiosceince FoxP3/transcription factor staining buffer set, and stored in 2% paraformaldehyde until analysis.<br><br>For the infected BMDMs: BMDMs were isolated from C57BL/6 mice by surgically removing the femurs and tibias, sterilizing the bone exterior with 70% ethanol, and removing the bone marrow by flushing with PBS. Red blood cells were lysed hypotonically with ACK lysis buffer and the remaining cells were cultured in DMEM with 10% hiFBS, 1%Pen/Strep, and 20 ng/mL rM-CSF. BMDMs were infected for 3 hours by inoculating media (without Pen/Strep) with bacteria and incubating at 37 degrees Celcius. For mitochondrial polarization and ROS detection: the infected cells were incubated for 1 hour in culture media supplemented with 500 ng/mL gentamicin, then stained, washed, and immediately analyzed.

	For the infected THP-1 cells: THP-1 cells were cultured in RPMI supplemented with 10% fetal bovine serum and infected for 3 hours by inoculating the media with bacteria and incubating at 37 degrees Celcius. For mitochondrial polarization and ROS detection: the infected cells were incubated for 1 hour in culture media supplemented with 500 ng/mL gentamicin, trypsinized with TripleExpress, stained, and immediately analyzed. For IRG1 quantification, the infected cells were incubated overnight in culture media supplemented with 500 ng/mL gentamicin, trypsinized, fixed, permeabilized, and intracellularly stained as described above, and then stored in 2% paraformaldehyde until analysis.
Instrument	A BD LSRII was used for the analysis of the mouse immune cell populations as well as IRG1 quantification in mouse immune cells and a BD FACSCantoII was used for the analysis of THP-1 and BMDM mitochondrial depolarization and ROS detection.
Software	BD FACSDiva v9 was used for data acquisition and FlowJo v10 was used for data analysis.
Cell population abundance	No post-sort fractions were collected in this study.
Gating strategy	For the infected mouse BAL and lung samples: cells were gated using forward scatter and side scatter, single cells were gated using forward scatter (area and height), live cells were gated using forward scatter and DAPI, and CD45+Epcam- immune cells were gated using AF700 and FitC; within the CD45+Epcam- cell population, SiglecF+CD11b+/- cells were identified using APC-Cy7 and AF594 and further gated identify SiglecF+CD11c+ alveolar macrophages using APC-Cy7 and Bv605, while SiglecF-CD11b- cells were further gated as CD11c- cells using Bv605 ,and then as Ly6G-Ly6C+/- monocytes or Ly6G+Ly6C +/- neutrophils using Bv421 and PerCP-Cy5.5. For the infected THP-1 and BMDM samples: cells were gated using forward scatter and side scatter, single cells were gated using forward scatter (area and height), and live cells were gated using DAPI; then IRG1 expression was assessed using the AF647 signal, or mitochondrial polarization and ROS were assessed using APC and PE. Boundaries between positive and negative staining were determined using unstained samples.

☒ Tick this box to confirm that a figure exemplifying the gating strategy is provided in the Supplementary Information.
